# Toxic metals in Loggerhead sea turtles (*Caretta caretta*) stranded freshly dead along Sicilian coasts

**DOI:** 10.1080/01652176.2023.2169781

**Published:** 2023-01-20

**Authors:** Gaetano Cammilleri, Francesco Giuseppe Galluzzo, Andrea Pulvirenti, Licia Pantano, Vittorio Calabrese, Antonino Gentile, Valentina Cumbo, Andrea Macaluso, Vito Macaluso, Antonio Vella, Vincenzo Ferrantelli

**Affiliations:** a Istituto Zooprofilattico Sperimentale della Sicilia “A. Mirri”, Palermo, Italy; b Dipartimento di Scienze della Vita, Università degli studi di Modena e Reggio Emilia, Modena, Italy; c Dipartimento di Scienze Biomediche e Biotecnologiche, Università degli Studi di Catania, Catania, Italy

**Keywords:** *Caretta caretta*, Loggerhead sea turtle, Sicily, heavy metals, lead, cadmium, arsenic

## Abstract

**Background:**

The Loggerhead sea turtle (*Caretta caretta*) is a marine reptile belonging to a monophyletic group of chelonians. As these animals are long-lived, they have the ability to accumulate pollutants.

**Aim:**

To collect epidemiological data on toxic metals in marine Loggerhead sea turtles.

**Materials and Methods:**

Forty Loggerhead sea turtles comprising 25 males and 15 females stranded freshly dead between 2013 and 2018 along the coasts of Sicily, Southern Italy, were examined for arsenic, cadmium, and lead accumulation in muscle and adipose tissues by means of a validated ICP-MS method. A modified K index as a growth condition factor, namely Fulton’s K index, was used. Samples were tested in duplicate. A Wilcoxon rank sum test was carried out to evaluate metal contents differences between muscle and adipose tissues and between genders.

**Results:**

The Fulton’s K index suggested a good body condition of the *C. caretta* recovered with mean values of 5.34 ± 3.40 (*n* = 40; ±SD). Detectable concentrations of lead were found in 70% of the samples analysed with mean values of 0.65 ± 1.67 mg/kg wet weight and 0.51 ± 1.29 mg/kg wet weight in muscle and adipose tissues, respectively. No significant differences in arsenic, cadmium, and lead were detected between genders. In addition, no significant correlation was found between modified K index and concentrations of arsenic, cadmium, and lead.

**Clinical relevance:**

Findings on muscle and adipose tissues suggest chronic exposure of *Caretta caretta* to high concentrations of especially lead which might negatively affect health and welfare of these marine turtles although body condition was good.

## 1. Introduction

The pollution by toxic metals in the aquatic environment has given rise to much discussion because of the risk of toxicity and bioaccumulation in the marine food chain (Sakai et al. 2000; Gochfeld 2003; García-Fernández et al. 2009; Jerez et al. 2010; Novillo et al. 2017). Natural and anthropogenic sources, including industrial and domestic sewage, and atmospheric deposits account for sources of toxic metals in the aquatic ecosystem (Slooff 1982; Sankar et al. 2006; Fazio et al. 2014; Cammilleri et al. 2018). Most of the waste accumulated in the sea comes from land and inevitably interacts with marine organisms (Galgani et al. 2014). The toxic metals accumulated in living organisms reflect the degree of environmental pollution (Canbek et al. 2007). The Loggerhead sea turtle (*C. caretta*) is a marine reptile belonging to a monophyletic group of chelonians containing seven extant species adapted to live in the marine environment, with a wide distribution in the Mediterranean Sea (Naro-Maciel et al. 2008). *C. caretta* is a long living vertebrate species that behave as carnivorous generalist feeders foraging principally on fish, crustaceans, molluscs, and other invertebrates (Godley et al. 1997). When turtles become adults, they return to natal beaches to reproduce and feed on coastal benthic habitats (McClellan and Read 2007). The most important areas of reproduction are Greece, Turkey, Libya, and Cyprus (Nagelkerken et al. 2003), whereas the most important feeding areas are the Tunisian continental platform and the Adriatic and Ionian seas (Mingozzi et al. 2008). In Italy, the most important nesting areas are the Ionic part of southern Calabria; smaller sites are located in the Pelage Islands and Southern Sicily (Mingozzi et al. 2008). *C. caretta*, according to its biological factors (i.e. age, gender, migration habits), could bioaccumulate toxic inorganic elements and compounds from contaminated food, marine sediment, and seawater (Cortés-Gómez et al. 2017). High concentration of trace elements in Loggerhead sea turtles could result in permanent physiological dysfunctions, however there are no data on the toxicity thresholds (Finlayson et al. 2016; Cortés-Gómez, Romero, et al. 2018; Canzanella et al. 2021; Cortés-Gómez et al. 2021).

Toxicological effect of toxic metals include cell death *via* apoptosis and necrosis, modification in the fertilization and survival of the eggs, immunosuppression and, skeletal malformations (García-Fernández et al. 2009; Cortés-Gómez et al. 2017). Studies have verified the distribution and evasion flux of toxic elements at the atmosphere/sea interface in Sicilian coasts (Southern Italy), illustrating that the intense industrial activity of the past has led to high pollution in these areas (Ministero ambiente 2008; Copat et al. 2012).

Arsenic (As) occurs in seawater mainly as inorganic arsenic in the trivalent and pentavalent states. Many algae can accumulate and biotransform inorganic arsenic to arsenosugars (Llorente-Mirandes et al. 2010). Regarding lead (Pb), the slowing down of aerosol Pb concentration decrease in the troposphere, is related to the implementation of antipollution policies and the limitation of Pb use as an additive in gasoline in the Mediterranean water column (Nicolas et al. 1994; Migon et al. 2008). The concentration of cadmium (Cd) in the marine environment is low, but it tends to accumulate in living organisms perturbing embryonic development and triggering stress protein synthesis, synthesis of globulins, reduce red blood cell production, and induce detoxification genes (Rie et al. 2001; García-Fernández et al. 2009; Komoroske et al. 2011; Camacho et al. 2013; Perrault et al. 2017; Cortés-Gómez, Tvarijonaviciute, et al. 2018). Polluted Cd areas were revealed in the Mediterranean sea around Sicily island, with concentrations comprising between 0.56 × 10^−3^ and 80.4 × 10^−3 ^μg/L (Censi et al. 2002). At present, few studies are focused on the presence of these elements in Mediterranean sea turtles and most of them did not investigate the correlation between growth parameters and bioaccumulation of these elements considering that these animals are long-lived and have the ability to accumulate pollutants (Maffucci et al. 2005; Storelli et al. 2008). Recent studies have verified a possible relation between growth condition factor, such as Fulton’s K condition indices, and the bioaccumulation of xenobiotics in aquatic organisms but the latter have focused only on organisms of commercial interest, not considering organisms useful in environmental monitoring (Mohamed Harris et al. 2019).

In this study, we examined Loggerhead sea turtles stranded on the Sicilian coasts (south Mediterranean Sea) for the detection of arsenic (As), cadmium (Cd), and lead (Pb) by an Inductively Coupled Plasma-Mass Spectrometry (ICP-MS) method. We analysed their distribution and accumulation in adipose and muscle tissues of this species and their correlation with morphometric parameters. Epidemiological data on the presence of toxic metals in South Mediterranean ecosystems were obtained.

## 2. Materials and methods

### 2.1. Sampling

**Figure 1.**
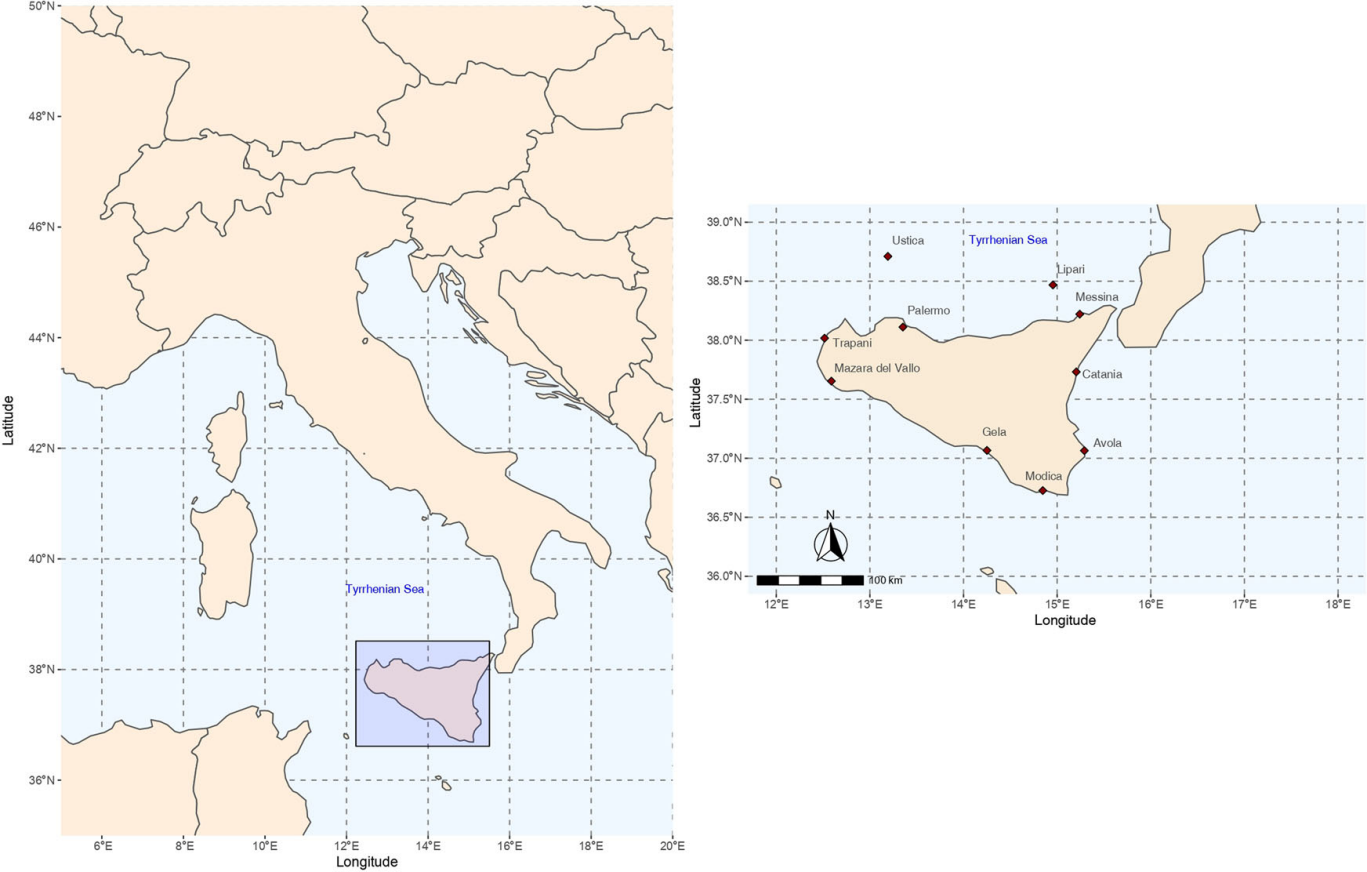
Map of the recovering sites of the loggerhead turtles (*Caretta caretta*) stranded between 2013 and 2018 in coastal areas of Sicily, Southern Italy.

Forty freshly dead *C. caretta* turtles stranded along 18 localities of the Sicilian coasts were recovered by the National Center of Turtles and Cetaceans Recovery from 2013 to 2018. The recovering sites (Palermo, Trapani, Mazara del Vallo, Gela, Modica, Avola, Catania, Messina, Lipari, Ustica) are shown in Figure 1. All the specimens were transferred to the Istituto Zooprofilattico Sperimentale della Sicilia (*via* Gino Marinuzzi 3, 90129, Palermo, Italy) and subjected to necropsy, biometry (straight carapace length, curved carapace length, etc.) and gender assessment by visual examination (Wyneken 2001; Casale et al. 2005). A modified Fulton’s K index was calculated with each loggerhead turtle recovered by the formula:

$$(\frac{\mathrm{body}\;\mathrm{mass}}{{\mathrm{SCL}}^{3}})*\;10^{4}$$

where SCL is the straight carapace length (cm) and body mass is expressed in Kg (Bjorndal et al. 2000). Muscle from limbs and adipose tissue from carapace depots were collected during necropsies and stored at −20 °C until chemical analysis. All the turtles were recovered freshly dead, with no visual evidence of tissue decomposition (Wyneken 2001; Heithaus et al. 2007). The morphometrics of the *C. caretta* specimens examined are summarised in Table 1. Initial inspections allowed us to identify the gender of the specimens examined, revealing 25 males and 15 females.

**Table 1.** Biometric parameters of the 40 specimens of *Caretta caretta* sampled.

	N	Mean ± SD	Min	Max
Weight	40	22.75 **±** 11.26	10.5	52
SCL	40	51.21 **±** 11.56	28	74.5
CCW	40	41.66 **±** 9.83	24	59
Modified K	40	6.03 **±** 3.58	2.55	18.72

Body weight (kg), SCL (straight carapace length, cm) and CCW (curved carapace width, cm) are shown.

### 2.2. Reagents and standards

All solutions were prepared with analytical ultra-pure grade reagents. Water for chromatography LiChrosolv ® was purchased from Merck KgaA (Darmstadt, Germany). Ultrapure nitric acid 60% was purchased from Merck KgaA (Darmstadt, Germany). Standard solutions: the multielement calibration solutions (As, Cd, Pb) were prepared at different concentration levels (0.001 - 50 µg/L) from 1000 mg/L single element ICP-MS grade standard from VWR International LTD (Randon, Pennsylvania, USA). A tuning solution for ICP-MS, capable of covering a wide range of masses (Ce, Co, Li, Mg, Tl, and Y 1 µg/l) were purchased from Agilent Technologies (Santa Monica, CA, USA) to optimize the performance of ICP-MS instrument before use. Internal standard solution: 100 mg/L standard stock solution of scandium (Sc), yttrium (Y), indium (In), terbium (Tb), rhodium (Rh), lutetium (Lu), lithium 6 (Li), indium (In), germanium (Ge) and bismuth (Bi) was purchased from Agilent Technologies (Santa Monica, CA, USA). Ultrapure grade carrier gas of argon, helium and hydrogen (99.9995% pure) were purchased from SOL S.p.A (Monza, Mi, Italy).

### 2.3. Analytical methods

The digestion process of the samples was carried out according to protocols reported before (Fazio et al. 2014; Cammilleri et al. 2020). Briefly, ∼1 g of the samples were transferred into previously decontaminated PTFE (poly-tetrafluoroethylene-tetrafluoroethylene) tubes with 3 ml of Ultrapure nitric acid 60% (V/V) and 5 mL of water. The sample digestion was carried out using a microwave digester Multiwave 3000 (Anton Paar, Graz, Austria). The digestion conditions for muscle and fat are reported in Table 2.

**Table 2.** Microwave digestion conditions for the muscle and fat samples analysed.

Phase	Power (W)	Ramp (min)	Hold (min)	FAN
1	600	10:00	40	1
2	0	0	15	3

#### 2.3.1. ICP-MS analysis

The elements were determined using a 7700x series ICP-MS (Agilent Technologies, Santa Monica, CA, USA). The sample extracted were pumped by a peristaltic pump from tubes arranged on an autosampler ASX-500 Series (Agilent Technologies, Santa Monica, CA, USA), combined with a quartz cyclonic spray chamber (water cooled at 2 °C). The instrumental settings and operative conditions can be found in Supplementary Material (S1).

A calibration curve based on 8 standard additions (BlankCal − 0.01 − 0.05 − 0.1 − 0.5 − 1 – 5 – 10 – 50 µg/L) was made to evaluate the linearity. A pool of digested samples was used for this test. The linearity of the calibration curve was considered acceptable for r^2^ > 0.999. The method limits of detection and quantification (LOD and LOQ) were calculated by the 3σ and 10σ approaches (ISO E 2005; Barwick et al. 2014). LOD is defined as the lowest concentration of an analyte in a sample that can be consistently detected with a stated probability (typically at 95% certainty), while LOQ is the lowest analyte concentration that can be quantitatively detected with a stated accuracy and precision (Armbruster and Pry 2008). Digested samples were used to calculate method LOD and LOQ according to protocols reported previously. The trueness of the method was assessed using the recovery of 3 concentration levels for each element (Fazio et al. 2014; Cammilleri et al. 2020). An acceptance limit between 90 and 110% was chosen (Barwick et al. 2014). A range of recovery between 97% and 102% was obtained for all the concentration levels of the analytes examined. Each sample was tested in duplicate.

### 2.4. Data collection and statistical analysis

All the results under the LOQ of the method were considered for the statistical analysis as half of the LOQ values (Hornung and Reed 1990; Lubin et al. 2004; Helsel 2005; Zota et al. 2014; Goniewicz et al. 2018). All the As, Cd, and Pb concentrations were expressed as wet weight (w.w.) and converted to dry weight (d.w.), using the mean water content (79%) for muscle according to Maffucci et al. (2005), for the comparison with other studies. The conditions of normal distribution and homogeneity of variances were not met. Therefore, a Wilcoxon rank sum test was carried out to evaluate As, Cd and Pb contents differences between muscle and adipose tissues and between genders with W as the Wilcoxon test statistic or the sum of the ranks in one of both groups and S as the sum of all squared rank differences. The relationship between the modified K index of the loggerhead turtles and toxic metals contents was examined *via* Spearman’s correlation method using the R®4.1.2 software (freeware available on https://cran.r-project.org/). The null hypothesis was rejected with a *p*-value less or equal than 0.05.

## 3. Results

The Modified Fulton’s K calculation suggested a good body condition of the *C. caretta* recovered, with mean values of 5.34 ± 3.40. The LOD-LOQ values for the analytes were: 0.001–0.002 mg/kg for As, 0.0008–0.001 mg/kg for Cd, and 0.002–0.006 mg/kg for Pb. The linear regression model was acceptable for all the analytes in the defined range, with a satisfactory correlation coefficient (r^2^ ≥ 0.999). The As, Cd, and Pb concentration (wet weight) of the loggerhead turtles examined are shown in Table 3.

**Table 3.** As, Cd, and Pb concentrations (mg/kg w.w.) detected in the tissues of the loggerhead turtles analysed.

Element	Sample type	N	>LOD	Mean ± SD (mg/kg)	Median	min-max (mg/kg)
As	Adipose tissue	40	8 (20)	0.69 ± 1.92	1.1	<LOD-7.5
	Muscle tissue	40	13 (32,5)	0.76 ± 1.79	1.78	<LOD-9.1
Cd	Adipose tissue	40	10 (25)	0.02 ± 0.05	0.07	<LOD-0.08
	Muscle tissue	40	15 (37,5)	0.07 ± 0.24	0.02	<LOD-1.3
Pb	Adipose tissue	40	18 (45)	0.51 ± 1.29	0.29	<LOD-6.12
	Muscle tissue	40	19 (47,5)	0.65 ± 1.67	0.35	<LOD-6.9

The fourth column shows the values of the samples above the limit of detection (>LOD).

**Figure 2.**
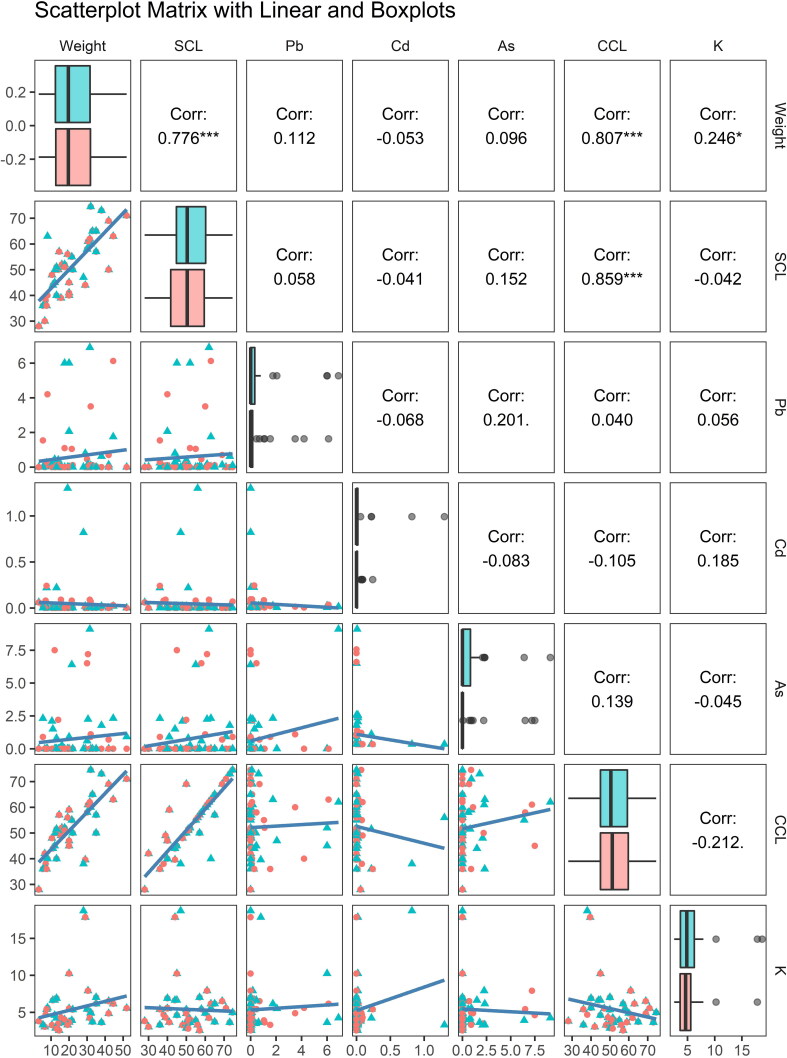
Boxplot, scatterplot, and correlation plot of the morphometric parameters reordered and As, Cd, Pb concentrations sorted by matrices (muscle in red and fat in blue obtained from 40 specimens). SCL = straight carapace length; CCL = curved carapace length. In the scatterplots: triangles = muscle, circle = adipose tissues. In the main diagonal the boxplot divided by group (muscle in red, fat in blue). The six-scatter plot below the principal diagonal showed a linear correlation SCL and weight, CCL and weight, SCL and CCL. The Spearman correlation coefficients between each pair of variables is above the principal diagonal. The correlation between weight and SCL is 0.805 (first row, second column) and supports the conclusion that as SCL increases, weight increase. No correlation was found between trace elements and the other parameters.

Toxic metal concentrations were detected in 13 (32.5%), 15 (37.5%) and 19 (47.5%) muscle samples for As, Cd and Pb, respectively. The muscle tissue of the Loggerhead sea turtles showed the highest occurrence and mean concentrations examined. However, no significant differences were found between adipose and muscle tissues for all the analytes examined (*W* = 11.5, *p*-value = 0.641 for Pb; *W* = 5, *p*-value = 0.381 for As; *W* = 4.5, *p*-value = 0.2555 for Cd). Initial inspections allowed us to identify the gender of the specimens examined, revealing 25 males and 15 females. Pb, Cd, and As were mostly found in male specimens, showing mean concentrations of 0.68 ± 1.83 mg/kg, 0.11 ± 0.24 mg/kg, and 1.05 ± 2.46 mg/kg, respectively in the muscle samples. However, no significant differences were found between males and females for all the toxic metals analysed (*W* = 8, *p*-value = 0.8831 for Pb; *W* = 6, *p*-value = 0.872 for Cd; *W* = 3, *p*-value = 0.3333 for As). The highest Cd concentration (1.3 mg/kg) was found in a muscle sample of a male loggerhead turtle of 19.2 kg stranded along the coasts of Catania. Instead, the highest concentration of Pb (6.90 mg/kg) and As (9.1 mg/kg) were detected in the muscle tissue of a male turtle of 31.5 kg stranded along the coasts of Milazzo. No significant correlation was found between modified K index and the analytes (*S* = 22,433, *p*-value = 0.163 for Pb, *S* = 26,493, *p*-value = 0.7483 for As, *S* = 28,271, *p*-value = 0.8855 for Cd; Figure 2).

## 4. Discussion

The results of this work showed elevated concentrations of Pb in 47.5% of muscles and 45% of adipose tissue of the Loggerhead sea turtles examined, revealing a chronic exposure to higher concentrations of this element compared to specimens from the Adriatic Sea (Eastern Mediterranean; below detection limit-0.09 mg/kg w.w.) (Storelli et al. 2005; Andreani et al. 2008), from East Spain coasts (0.04 ± 0.05 mg/kg w.w.) (Jerez et al. 2010), and Southern coasts of the Tyrrhenian sea (below detection limit-0.11 mg/kg w.w.) (Esposito et al. 2020). Our findings are surprising if one considers the regulation that limited the leaded gasoline consumption applied in European countries since the 1970s (Nicolas et al. 1994; Tian and Ruiz-Pino 1995) and seem to disagree with what was assumed by Jerez et al. (2010) regarding a reduction of Pb concentrations in the Mediterranean. On this basis, a statistical simulation of the surface and bottom layers of the Mediterranean carried out by Tian and Ruiz-Pino (1995) predicted an increase in Pb concentrations in the Eastern Basin, confirmed by the high concentrations of this element found in small pelagic fish and other marine organisms (Tigano et al. 2009; Copat et al. 2012). Furthermore, a clear distinction between the anthropogenic inputs of lead on the Sicilian and Murcia coasts should be considered (Ministero ambiente 2008; Jerez et al. 2010; Copat et al. 2012).

The main sources of Pb pollution in Sicilian coasts are attributable to refining activities and/or oil leaks in the Augusta roadstead; presence of numerous chemical and petrochemical industrial plants, refineries, as well as a purification plant for the disposal of industrial and civil wastewater located along the Sicilian coasts (Cosentino et al. 2013; Romano et al. 2013, 2016). Augusta roadstead (East Sicily, Italy) represents one of the largest European petrochemical poles that is considered highly contaminated by several industrial discharges that were reversed directly into the sea when environmental legislation was not active (Rothwell and Croudace 2015; Benedetti et al. 2022).

This has inevitably led to a continuous and prolonged supply of pollutants to the sea over time, both through the aquifer and the numerous discharges (Ministero ambiente 2008). Most metals, including Pb, are not subject to bacterial degradation. Therefore, they are an essentially permanent addition to marine ecosystems. Consequently, they accumulate in the sediments and are bio magnified up the food chain, with the top predators receiving the most significant dose of conservative substances (Kirpichtchikova et al. 2006). On this regards, our Pb results seem comparable with those found by Russo et al. (2003) in the muscle tissues of 10 juveniles and subadults *C. caretta* stranded along the Sicilian coasts, confirming the high Pb burdens in this area and the absence of correlation between the size of Loggerhead sea turtles and the accumulation of toxic elements. Although not directly comparable, our data follow previous studies on the bioaccumulation of toxic metals in other marine organisms sampled along the Sicilian coastline (Tigano et al. 2009; Copat et al. 2012). Furthermore, our results are similar to those detected in Loggerhead sea turtles from the northern Cyprus (Godley et al. 1999), confirming a greater exposure to this element in the South eastern Mediterranean.

The Pb concentrations observed in this work are higher than those found in other studies on Loggerhead sea turtles stranded on Atlantic French and Japanese coasts (Sakai et al. 1995; Caurant et al. 1999; Sakai et al. 2000), which revealed average values between 0.03 and 0.09 ± 0.03 mg/kg w.w. in muscle and adipose tissues. The high Pb concentrations found in this study could explain the lack of a significant distinction between muscle and fat tissue accumulation due to this species’ physiology, in particular, to the activation of metal detoxification mechanisms such as the expression of Metallothioneins (MTs). MTs play a special part in regulating the intracellular availability of metals. The binding by MT of xenobiotic metals in an organism protects it from their toxic effects (Jakimska et al. 2011). MTs are activated by metal regulatory transcription factor 1 (MTF1), present in both adipocytes and muscle cells (Morão et al. 2022). A lack of significant differences between muscle and fat samples for Cd and Pb was also found by Novillo et al. (2017) in *C. caretta* specimens from Spanish coasts. The epidemiological data obtained in this work could be helpful for further toxicological studies describing the threshold concentrations of Pb above which harmful effects would be likely, in accordance to what was stated by Novillo et al. (2017).

As opposed to Pb, the Cd concentrations detected in the muscle and adipose tissues of the Loggerhead sea turtles analysed in this work were slightly lower than those found in specimens analysed in the Adriatic Sea (Franzellitti et al. 2004; Storelli et al. 2005), Atlantic coasts of France (Caurant et al. 1999) and Japan (Sakai et al. 1995; 2000). The Cd mean concentrations found in this study were comparable to those found in muscle tissues of Loggerhead sea turtles stranded in Cyprus (Godley et al. 1999) but up to twenty times lower than loggerhead turtles stranded on Atlantic coasts (Torrent et al. 2004; Nicolau et al. 2017). No significant correlation was found between cadmium concentration and the morphometric parameters of the specimens examined, in contrast to what was reported by Storelli et al. (1998) but in accordance to the findings of Esposito et al. (2020) in *C. caretta* specimens from the Thyrrenian sea. Our results suggest that the body burden of cadmium in Loggerhead sea turtles is not mainly dependent on accumulation phenomena as described by Catsiki et al. (1994), also given that Cd is not bio-magnified (Gray 2002). The low concentrations of Cd could be mainly attributed to individual variations in diet. Squids are important cadmium accumulators and a source of this element for predators at the top of the marine food web, such as Loggerhead sea turtles (Bustamante et al. 1998; Storelli et al. 2005; Jerez et al. 2010). Contrary to Loggerhead sea turtles of other areas, which include squids in their diet, Loggerhead sea turtles in the eastern Mediterranean Sea feed upon organisms with low Cd concentrations, such as benthic molluscs and crustaceans (Godley et al. 1997).

Very low As concentrations were detected in this study, in contrast to what was found in Loggerhead sea turtles stranded in the South Adriatic Sea (15.47 ± 11.91 mg/kg w.w.) (Storelli et al. 1998; Storelli and Marcotrigiano 2000), Portugal (14.7 ± 1.47 mg/kg w.w.) (Nicolau et al. 2017) and Canarias (7.35 ± 1.37 mg/kg w.w.) (Torrent et al. 2004), even though the diet of southeast Mediterranean Loggerhead sea turtles is primarily based on small crustaceans which usually show high concentrations of total As (Norin et al. 1985; Godley et al. 1997). Our findings on As concentrations seem to be similar to what was found by Abdallah and Abd-Allah (2010) in Loggerhead sea turtles stranded on Egyptian coasts. Besides dietary differences, bioaccumulation of contaminants in marine biota can be viewed as a result of the interaction of numerous biological factors comprising age, trophic level, locality of sampling, and reproductive state (Storelli and Zizzo 2014; Cammilleri et al. 2017).

The toxic metal concentrations of the samples analysed did not differ between genders, suggesting that overall metal loads are similar for males and females. However, the lack of significant differences could be due to methodological limitations linked to the unbalanced number of males and females examined in this work. Maternal metal transfer has been documented in sea turtles (Ikonomopoulou et al. 2011), so the higher toxic metal concentrations found in males may result from females’ ability to offload a significant portion of the metal burden into their offspring offloading maternal transfer, is known for both oviparous and live-bearing reproductive strategies. Thus, as the female ages, it can offload its metals burden to her eggs, thereby decreasing her tissue concentrations. On the contrary, males do not have a similar offloading mechanism, so they continually accumulate metals; although not significantly different, this could explain the higher toxic metals values found in the male specimens examined. Differences in metal concentrations between genders may be related to age. For this purpose, further studies based on age determination by skeleton chronological analysis, coupled with morphometric analysis, are needed to verify the accumulation of these substances over time.

## 5. Conclusions

A reliable ICP-MS method was carried out to detect As, Cd, and Pb in muscle and adipose tissues of Loggerhead sea turtles stranded along the Sicilian coasts. To the best of our knowledge, the present work reported for the first time the presence of arsenic in loggerhead turtles stranded on the shores of Sicily (Southern Italy). About 48% of the samples analysed showed Pb concentrations over the LOD, with very high concentrations (up to 16 times the data reported in the literature). The results obtained confirmed that toxic metals principally accumulate in muscle tissue and showed lower Cd and As contents than reported in other studies. No significant differences were found between the genders. Our data also confirmed the possibility to monitor *C. caretta* as a bioaccumulator of toxic metals exposure in the aquatic environment thereby assessing the health of wildlife species. The findings presented here provide interesting epidemiological data to increase the knowledge of pollution impacts on marine turtles inhabiting contaminated areas.

## Supplementary Material

Supplemental Material
