# Supplementary material for: Toxic metals in Loggerhead sea turtles (Caretta caretta) stranded freshly dead along Sicilian coasts
Source: Vet Q. 2023 Jan 20;43(1):1–10. doi: 10.1080/01652176.2023.2169781 (PMC9870007; doi:10.1080/01652176.2023.2169781)
Supplement: Supplemental Material [file TVEQ_A_2169781_SM7024.docx]

**Toxic elements in loggerhead turtles (*Caretta caretta*) stranded along Sicilian coasts (South Mediterranean)**

Gaetano Cammilleri^a^, Francesco Giuseppe Galluzzo^b*^, Andrea Pulvirenti^b^, Licia Pantano^a^, Vittorio Calabrese^c^, Antonino Gentile^a^, Valentina Cumbo^a^, Andrea Macaluso^a^, Vito Macaluso^a^, Antonio Vella^a^, Vincenzo Ferrantelli^a^

^a^ Istituto Zooprofilattico Sperimentale della Sicilia “A. Mirri”; Via Gino Marinuzzi 3, 90129, Palermo, Italy

b Dipartimento di Scienze della Vita; Università degli studi di Modena e Reggio Emilia, Via Università 4, 41121 Modena, Italy

c Dipartimento di Scienze Biomediche e Biotecnologiche, Università degli Studi di Catania, Torre Biologica Via Santa Sofia, 95123 Catania, Italy

* Correspondence: 282446@studenti.unimore.it; +390916565258

Table S1. Instrumental parameters and the operative condition of the ICP-MS method.

| Parameter | Setting |
| --- | --- |
| RF-Power (W) | 1550 |
| Reflected power | < 5 |
| Carrier gas flow (mL/min) | 1.0 |
| Plasma gas flow (L/min) | 15 |
| Auxiliary gas flow (mL/min) | 1.0 |
| Spray chamber | Water cooled double pass |
| Spray chamber temperature (°C) | 2 |
| Lens voltage (V) | 6.25 |
| Mass range (amu) | 6 - 209 |
| Mass resolution | 0.7 |
| Integration time points/ms | 3 |
| Points per peak | 3 |
| Replicates | 3 |
| RF-Power (W) | 1550 |
| Reflected power | < 5 |
| Carrier gas flow (mL/min) | 1.0 |
| Plasma gas flow (L/min) | 15 |
| Auxiliary gas flow (mL/min) | 1.0 |
| Spray chamber | Water cooled double pass |
| Spray chamber temperature (°C) | 2 |
| Lens voltage (V) | 6.25 |
| Mass range (amu) | 6 - 209 |
| Mass resolution | 0.7 |
| Integration time points/ms | 3 |
| Points per peak | 3 |
